# Supplementary material for: Multi-Cohort Transcriptomic Subtyping of B-Cell Acute Lymphoblastic Leukemia
Source: Int J Mol Sci. 2022 Apr 20;23(9):4574. doi: 10.3390/ijms23094574 (PMC9099612; doi:10.3390/ijms23094574)
Supplement: Supplementary file 1 [file ijms-23-04574-s001.zip › supplement_figures_v8.pdf]

Supplement Figure S1

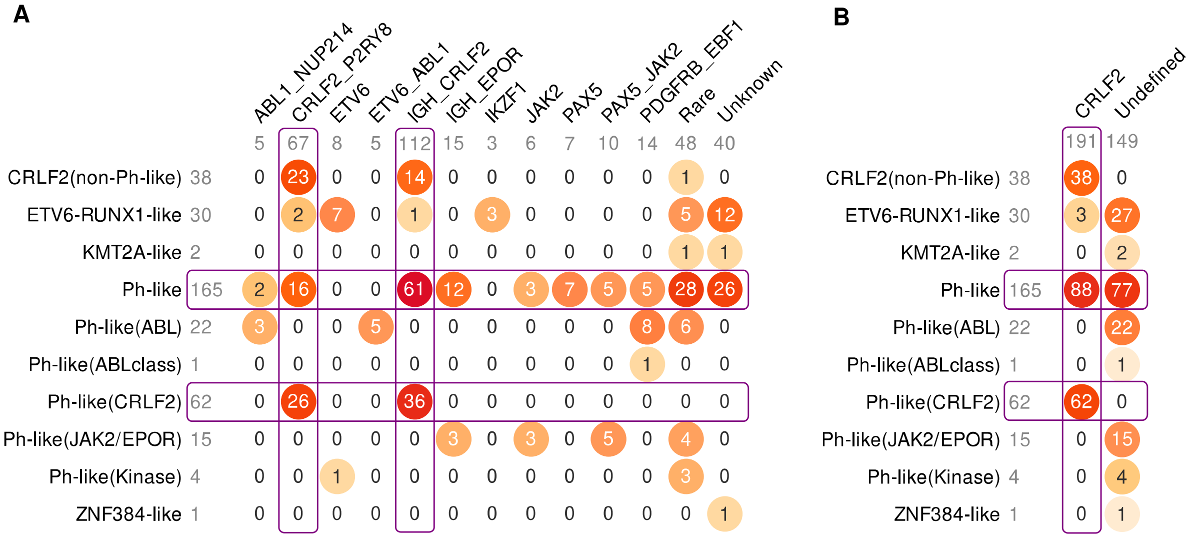

Re-definition of subtypes that were derived predominantly from clustering of gene expression data. From a machine learning perspective, it would be problematic to first define a subtype using gene expression profiles, and then predict the subtype using the same data (circular design). To preserve sound design for this study, the Ph-like subtype was not used as the target for prediction by RNA-seq data. **A**) Overlap between RNA-based "like" subtypes (vertical) and combinations of gene alterations (horizontal). **B**) Patients that were Ph-like or otherwise derived from RNA-seq clustering were assigned to the CRLF2 subtype if they harboured alterations involving the CRLF2 gene as this was the most frequent genetic lesion within these patients.

Supplement Figure S2

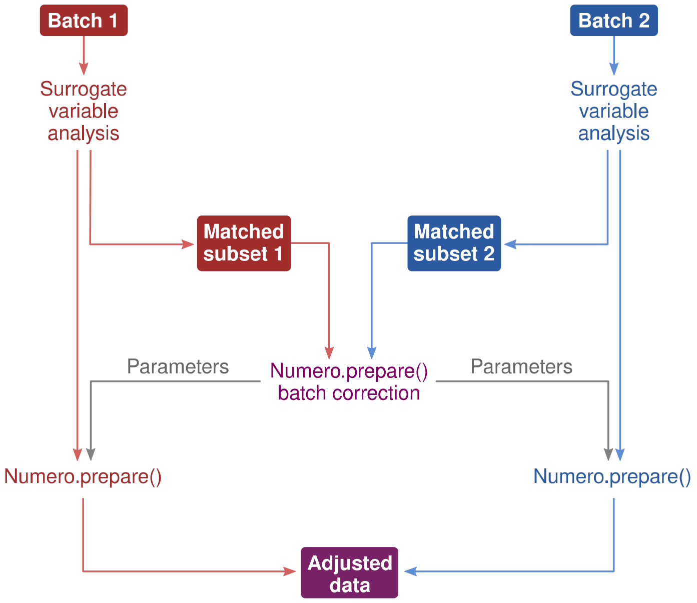

Schematic illustration of batch corrections. The RNA-seq datasets contained undesirable correlations between gene expression and multiple potential confounders including library strandedness, subtype prevalence across different locations and other cohort effects. To prevent the classification models from fitting to these patterns, we first applied surrogate variable analysis (SVA) to remove variation that was not related to the traits of interest within each batch. Note that applying SVA to the entire dataset could lead to over-optimistic classifiers, since it would amplify existing correlations between batch membership and the subtypes that arise from the cohort structure. To remove the correlations between batch membership and biological subtypes, we created matched subsets from each batch that had pair-wise identical age, sex, genetic subtype and known lesion profile. It thus became safe to do standard batch correction using the Numero library for these subsets. Lastly, the adjustment parameters from the subset analysis were used for adjusting the batch effects of the original data.

Supplement Figure S3

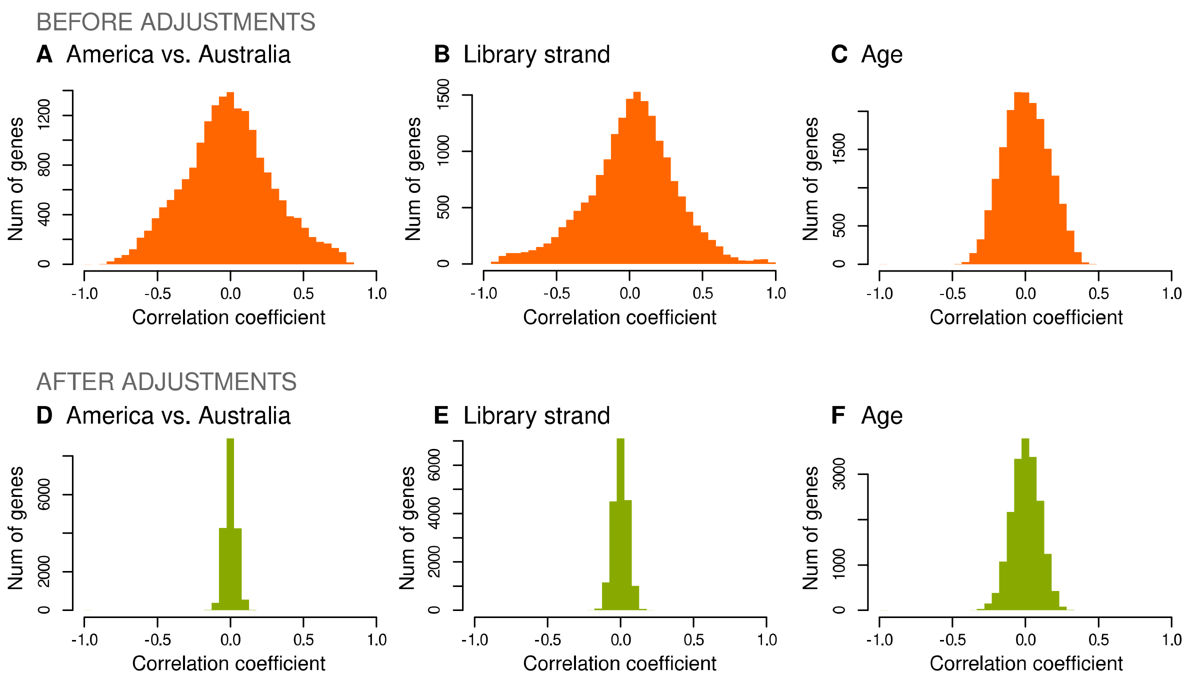

Comparison of Pearson correlations between log-transformed read counts and patient meta-data before and after batch correction. Age was not used as a matching criterion in those batch correction steps that involved comparisons between pediatric and non-pediatric batches.

Supplement Figure S4

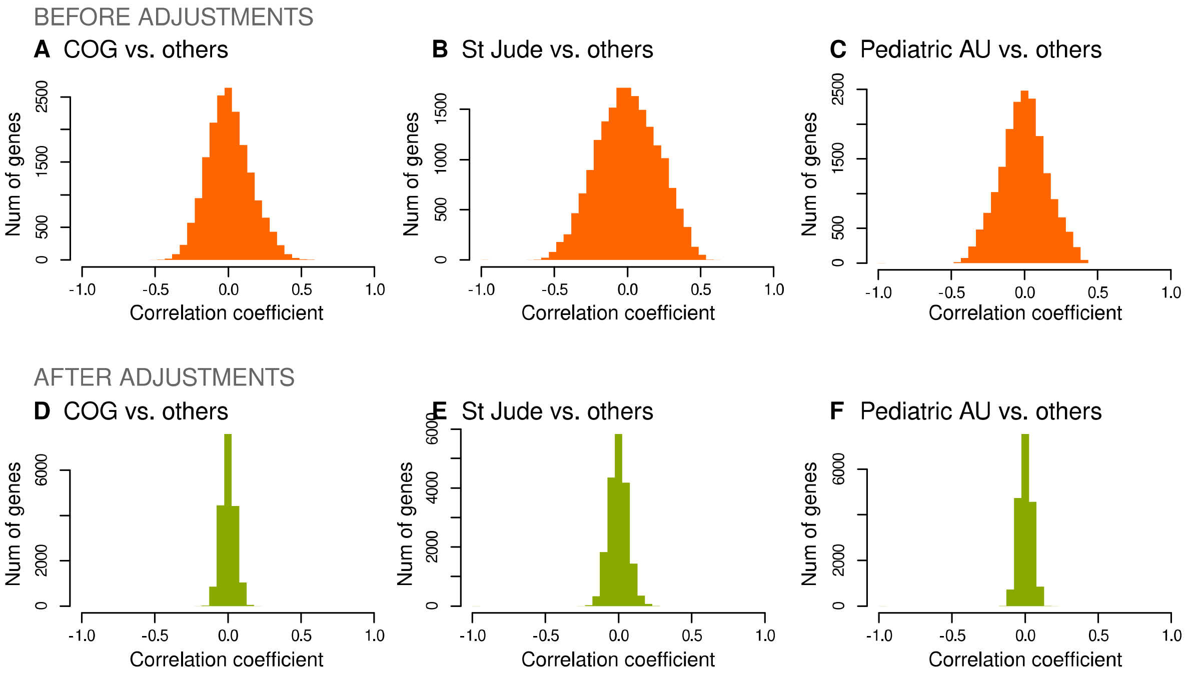

Comparison of Pearson correlations between log-transformed read counts and patient meta-data before and after batch correction. Age was not used as a matching criterion in those batch correction steps that involved comparisons between pediatric and non-pediatric batches.

Supplement Figure S5

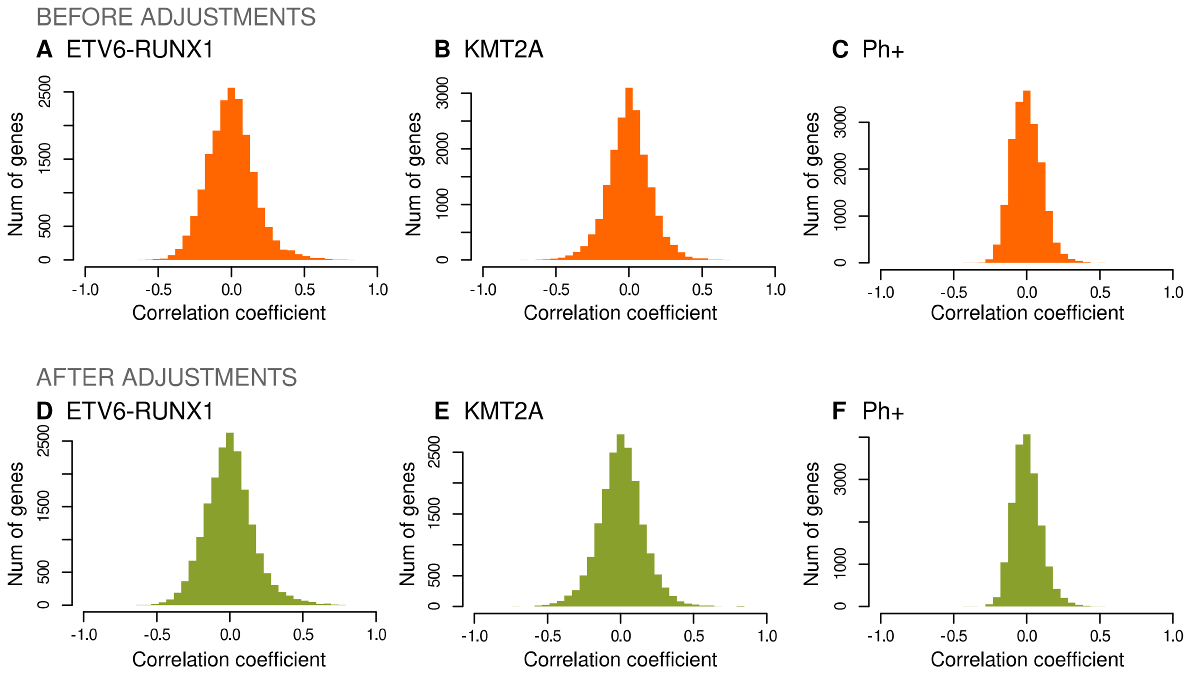

Comparison of Pearson correlations between log-transformed read counts and patient meta-data before and after batch correction.

Supplement Figure S6

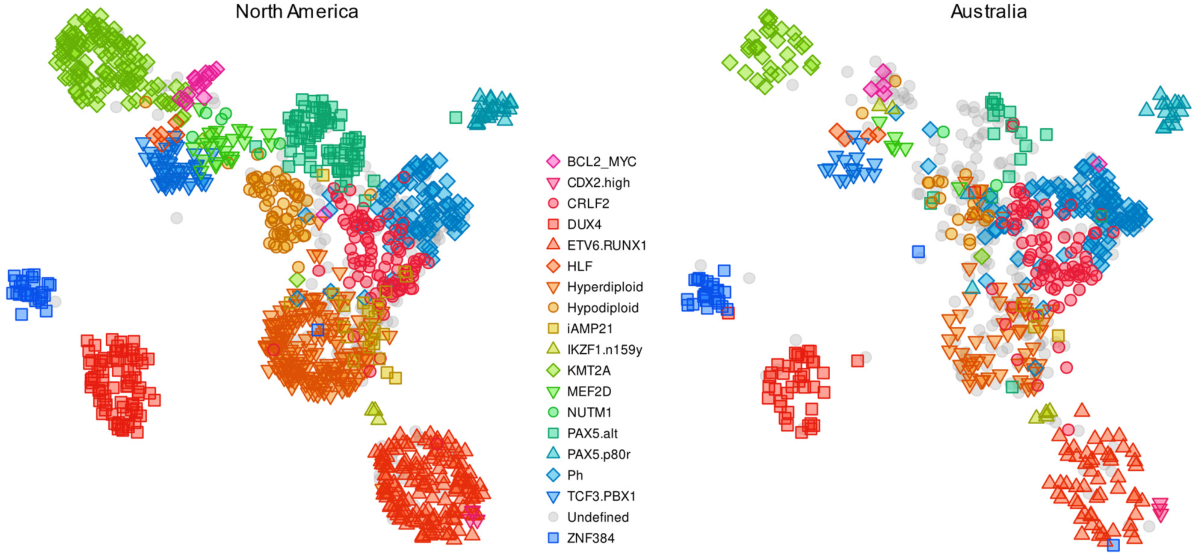

Uniform Manifold Approximation and Projection (UMAP) trained with batch corrected North American data and using genes that were optimised the centroid classifier. The scatter plot was produced by applying the UMAP to the unadjusted North American dataset and Australian datasets, respectively. The grey symbols represent undefined genetic subtypes.

Supplement Figure S7

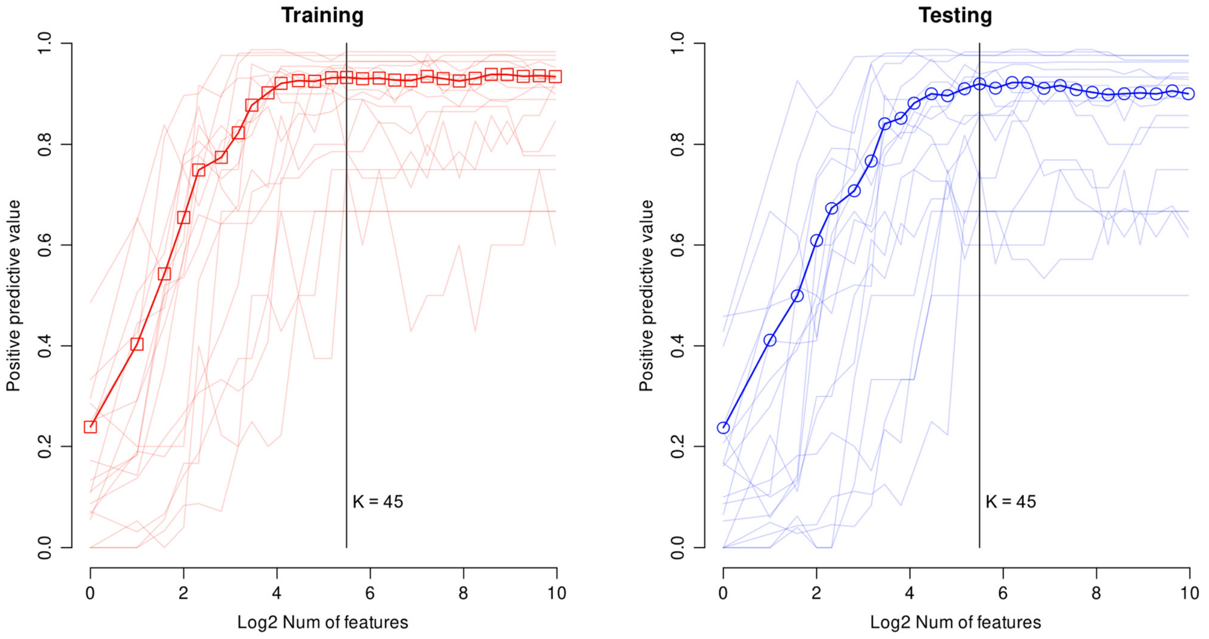

Optimization of a hyperparameter (number of input genes) for the centroid classifier. The model was trained with half the batch corrected North American dataset and tested with the other half. The curves depict overall positive predictive values that were calculated by applying the centroid models with different numbers of inputs to unadjusted North American data. We chose K = 45 as the final hyperparameter value.

Supplement Figure S8

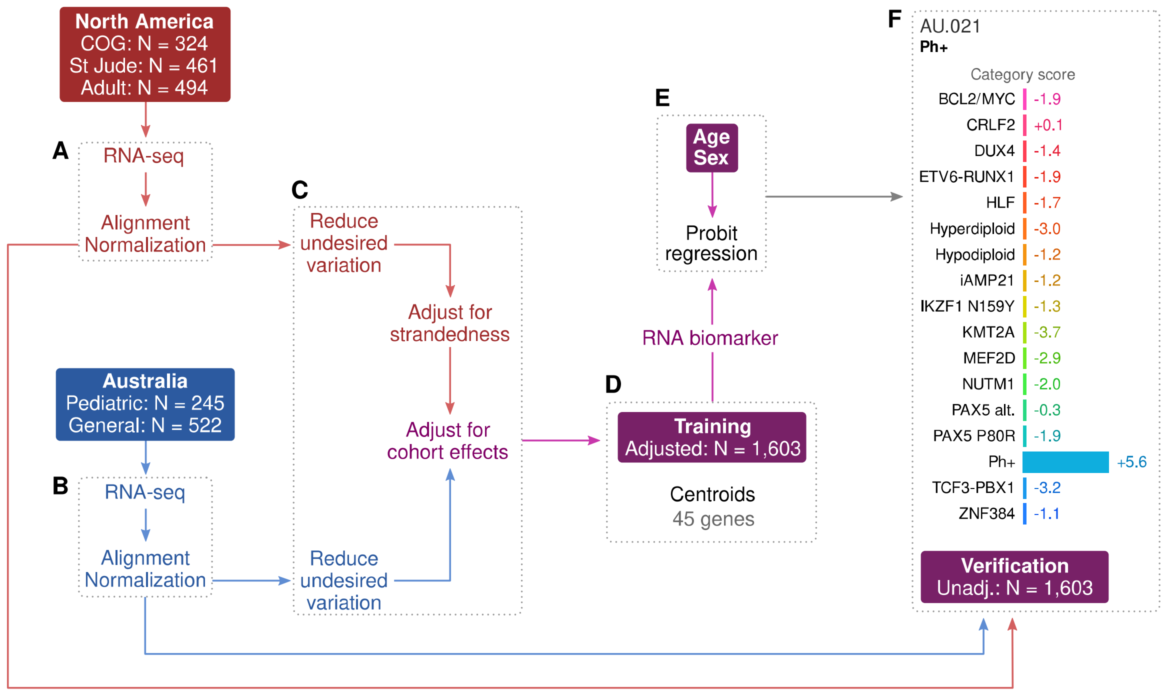

Centroid classifier as trained for the Allspice R library. All available samples that had a verified genetic subtype were used as a training set. Altogether 57 inputs out of 6,673 stable genes were prioritized according to the clumping algorithm described in Methods. The model was trained with batch corrected data and performance metrics were calculated from unadjusted expression levels.

Supplement Figure S9

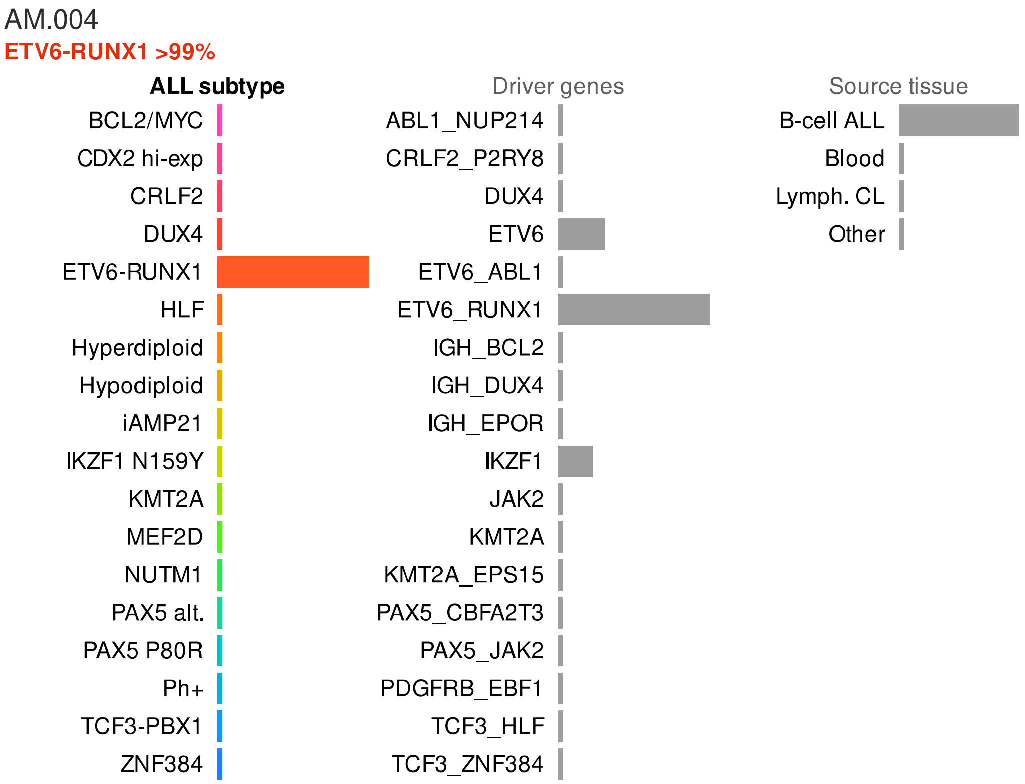

ETV6-RUNX1 case study from a North American cohort. This is an example of a patient with a distinct gene expression profile associated with a directly observable driver fusion.

Supplement Figure S10

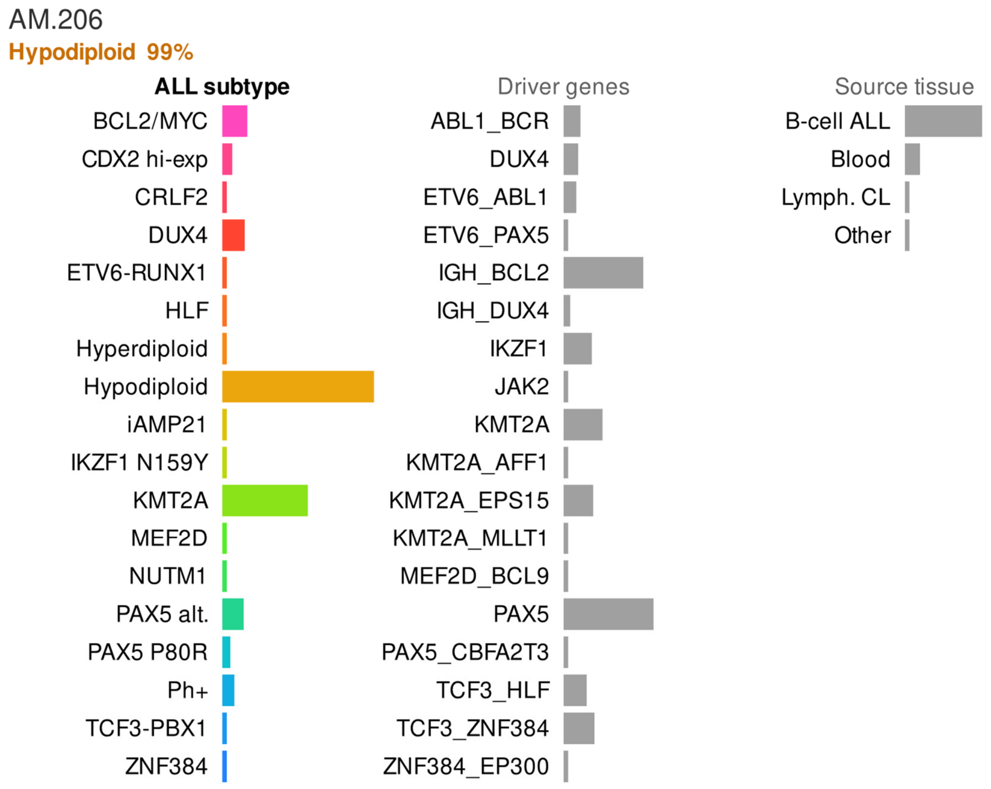

Hypodiploid case study from the North American dataset. Patients with chromosomal alterations are easy to detect via karyotyping, however, the extensive genetic alterations may affect multiple genes and pathways which may make it difficult to ascertain specific targets for molecular therapies. In this example, the RNA-seq profile suggests that the transcriptional consequences are compatible with a broad group of individuals that have BCL2, KMT2A and/or PAX5 lesions. The additional information from RNA-seq provides clues on the combination of genomic drivers that may be specific to this patient.

Supplement Figure S11

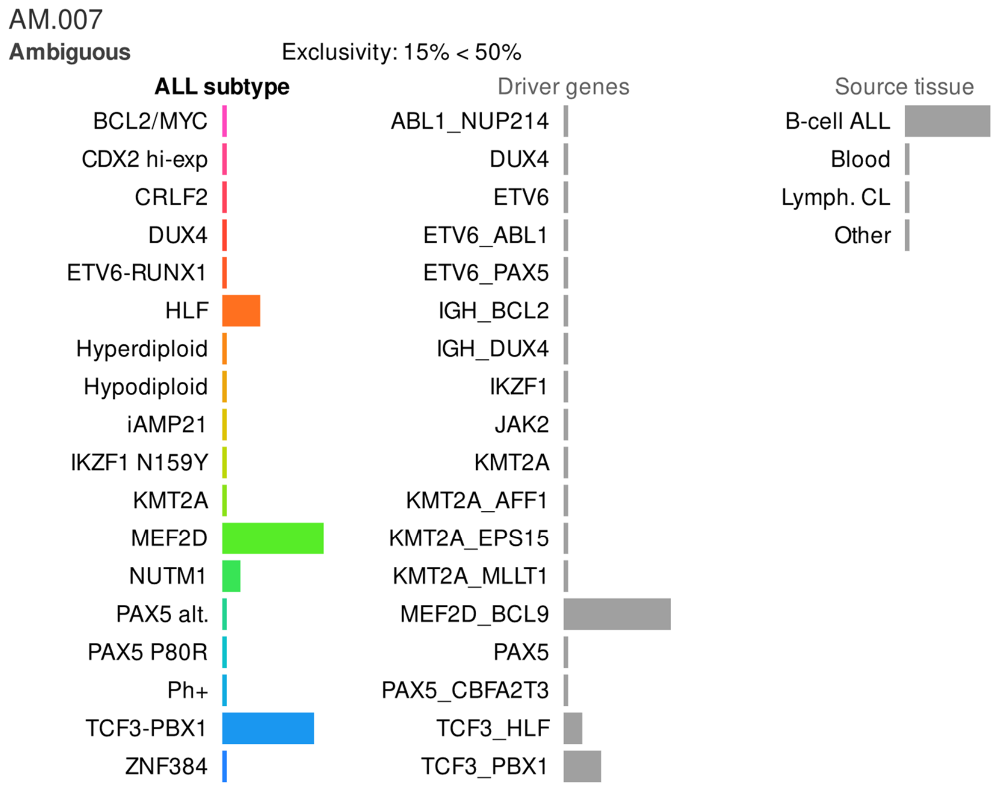

Ambiguous case study from the North American dataset. It is possible for a patient to exhibit multiple genetic lesions that drive ALL. Furthermore, the transcriptional consequences from different lesions may converge to similar profiles, which means that some patients will exhibit mixed gene expression characteristics. In this example, the transcriptional profile is in between the MEF2D and TCF3-PBX1 subtypes. The secondary analysis of driver genes (middle panel) suggests the combined lesion of MEF2D and BCL9 may be an important factor for this individual.

Supplement Figure S12

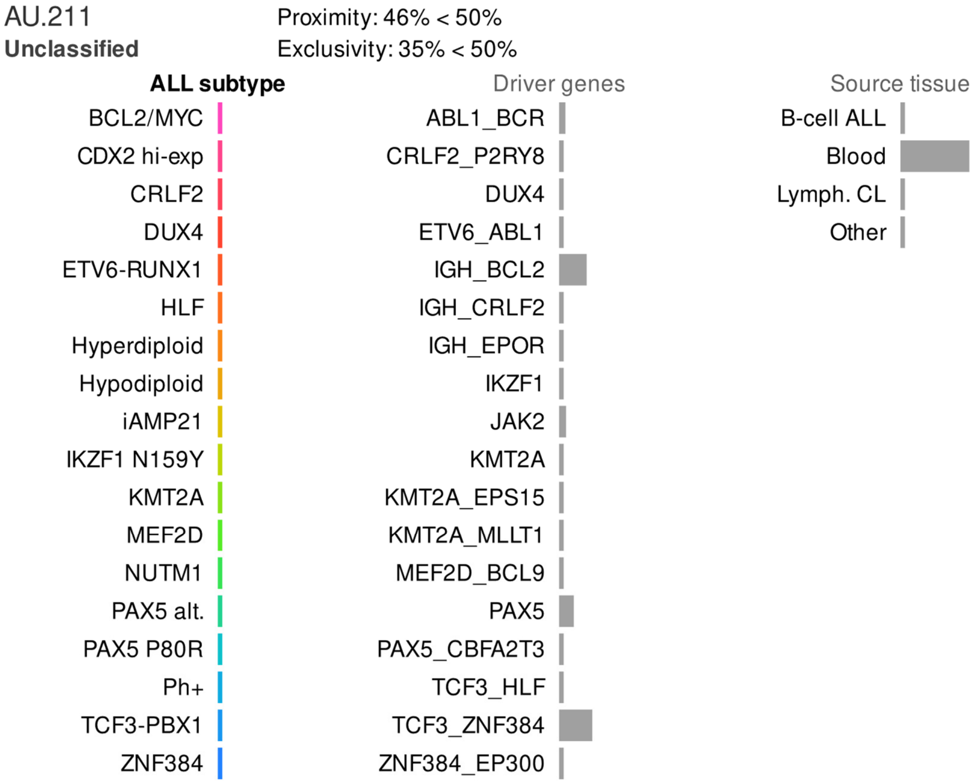

Unclassified case study from the Australian dataset. An unusual gene expression profile can represent a previously unknown subtype, however, it is more likely that the patient either had low leukemia burden or that incidental factors such as instrument failures or sample handling accidents had affected the sample quality. In Allspice, atypical samples are characterised by the lack of signals across the subtypes and genetic drivers. Here, there is also indication that leukemia burden may be low (see the tissue panel on the right).

Supplement Figure S13

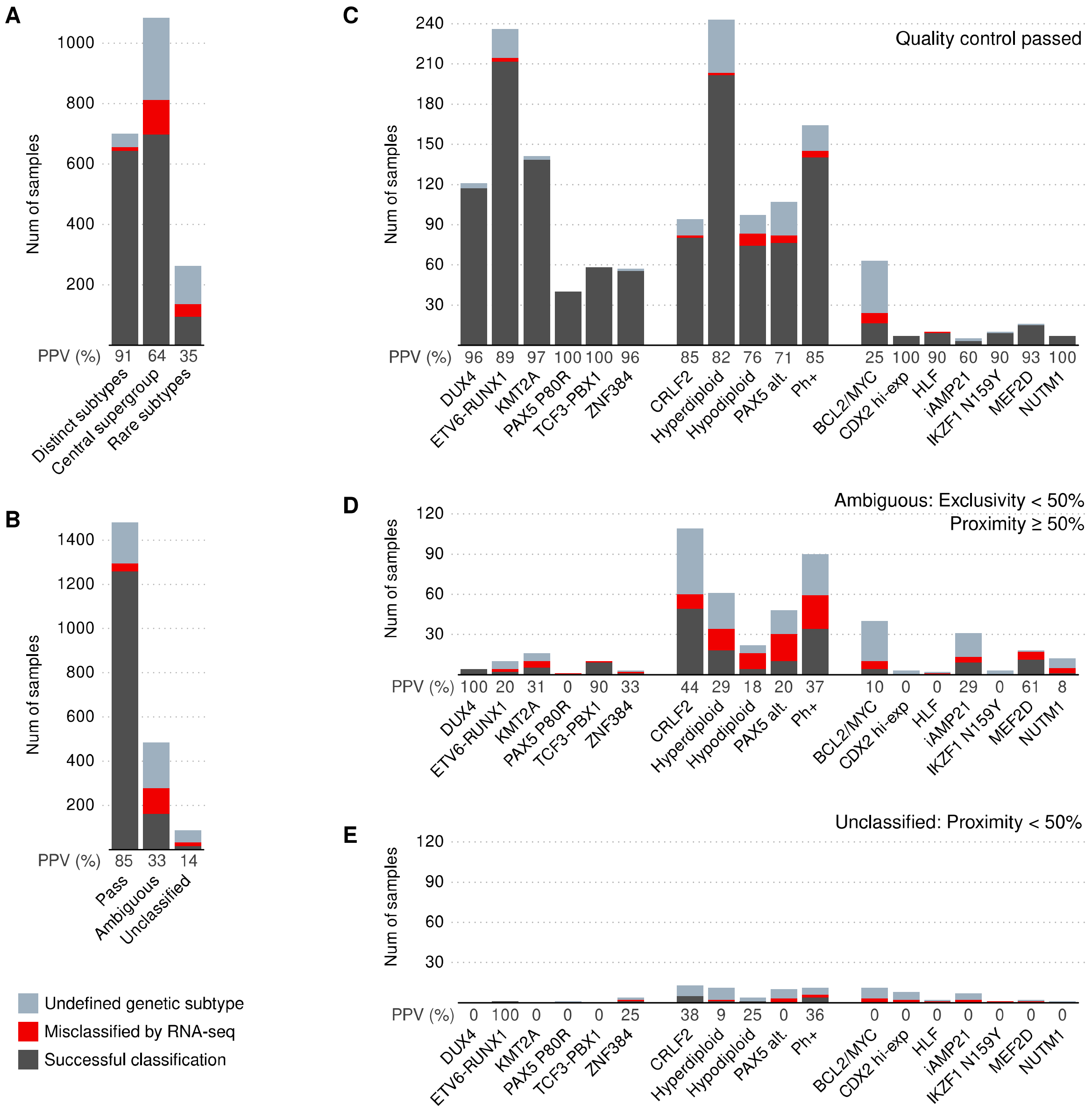

Detailed breakdown of the Allspice classifier performance with respect to subtypes and sample quality. **A**) Samples classified as one of the distinct subtypes exhibited high accuracy and a low proportion of undefined genetic subtypes. The central subgroup was less clear with lower accuracy overall. **B**) Samples that passed quality control (proximity ≥ 50%) were accurately classified. Ambiguous and unclassified samples included a high proportion of undefined subtypes. **C-E**) Samples classified by Allspice into specific subtypes, stratified into quality groups.

Supplement Figure S14

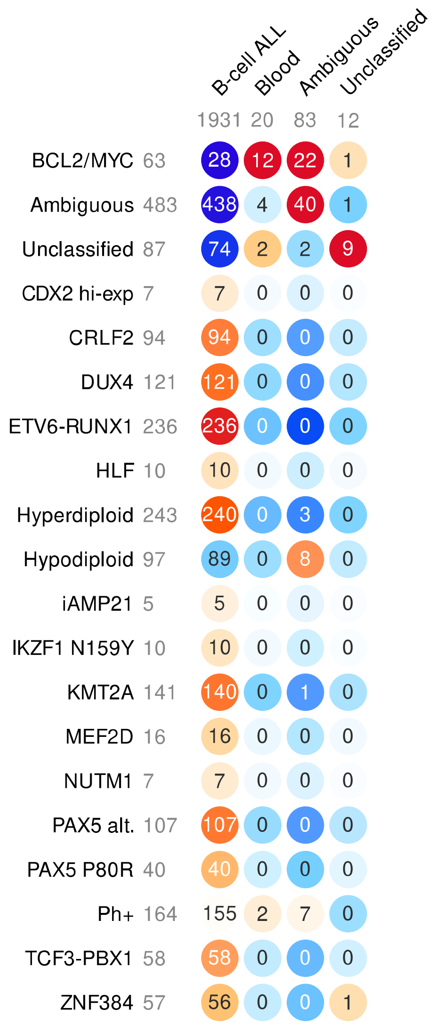

Application of a secondary tissue classifier contained within the Allspice package. The numbers of B-cell ALL samples are shown (total 2,046). The color intensity indicates deviation from the expected category distribution given the category sizes. Substantial classification of the ALL patient samples as whole blood or ambiguous source were observed for the BCL2/MYC subtype (35 out of 63).

Supplement Figure S15

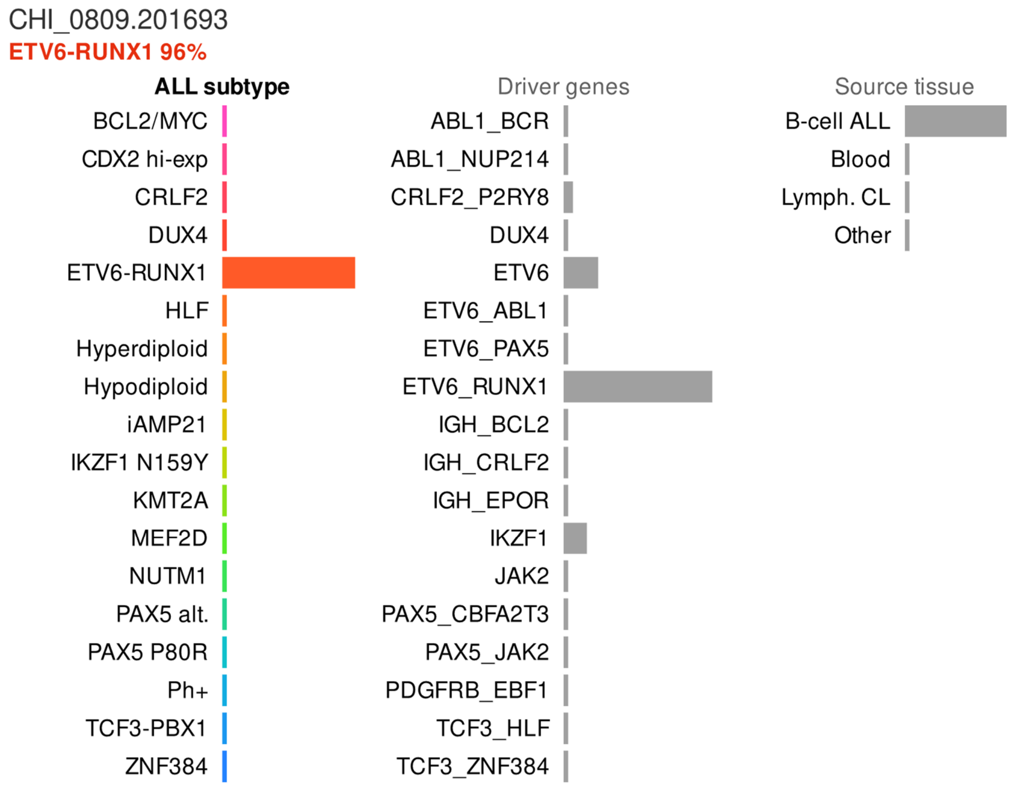

A case study of a young boy who was tested at our study center in South Australia. The sample arrived after Allspice was finished and it was not used anywhere else in this manuscript. No ETV6-RUNX1 fusion was identified by molecular genetics (Karyotype, FISH, SNParray) or within the RNA-seq data. RNA-seq identified the following fusions (appear to be consistent with complex 3-way translocation idicated by FISH): ETV6 (chr 12, exon 7) - HDAC9 (chr 7, exon 13), HDAC9-ETV6 (3’ UTR exon 13 - intron 1), UBE4B (chr 1, exon 2) - ETV6 (chr 12, exon 8), ETV6 (chr 12, exon 8) - IKZF1 (chr 7, exon 3). However, the transcriptional profile was consistent with ETV6-RUNX1 and the patient was subsequently classified as ETV6-RUNX1-like.

Supplement Figure S16

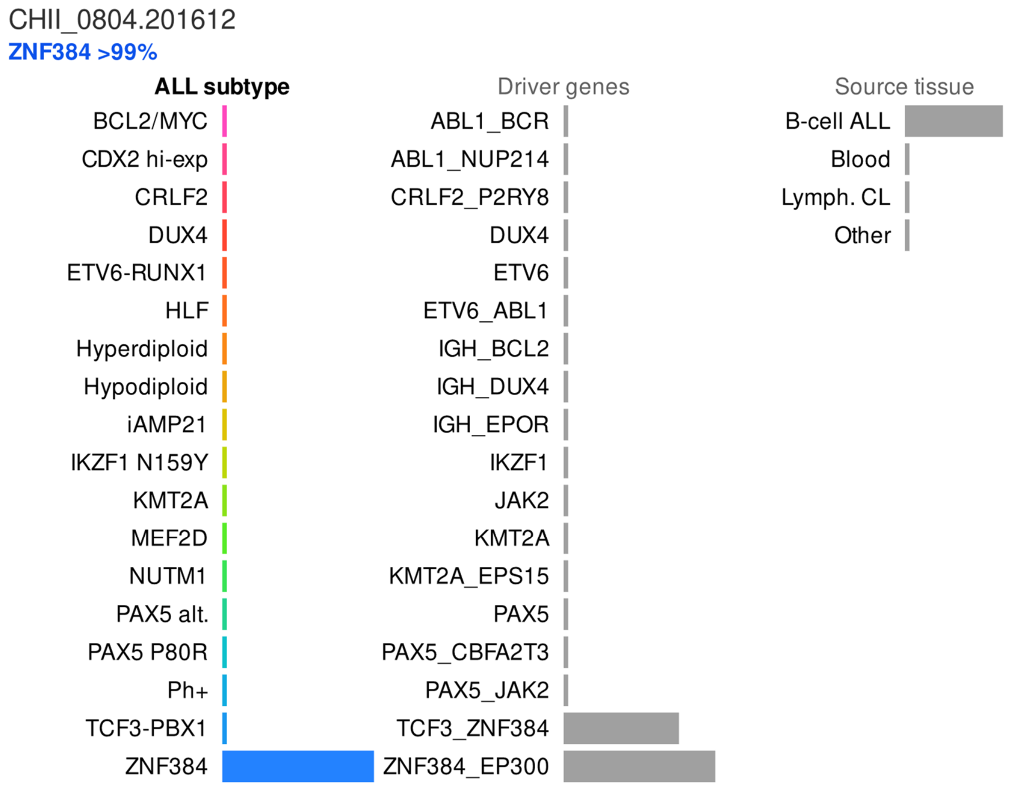

A case study of a girl who was tested at our study center in South Australia. The sample arrived after Allspice was finished and it was not used anywhere else in this manuscript. No known ZNF384 fusions identified by molecular genetics or RNA-seq. FISH performed with ZNF384 Break Apart Probe, no rearrangement found. Detailed search of sequence data identified an AHSA2-ZNF382 fusion called by 9 reads by fusion catcher only. In UCSC Genomic coordinates maps to intron 78 of USP34 and the intragenic region between ZNF383 and ZNF461. The patient was classified as ZNF384-like based on the transcriptional phenotype.

Supplement Figure S17

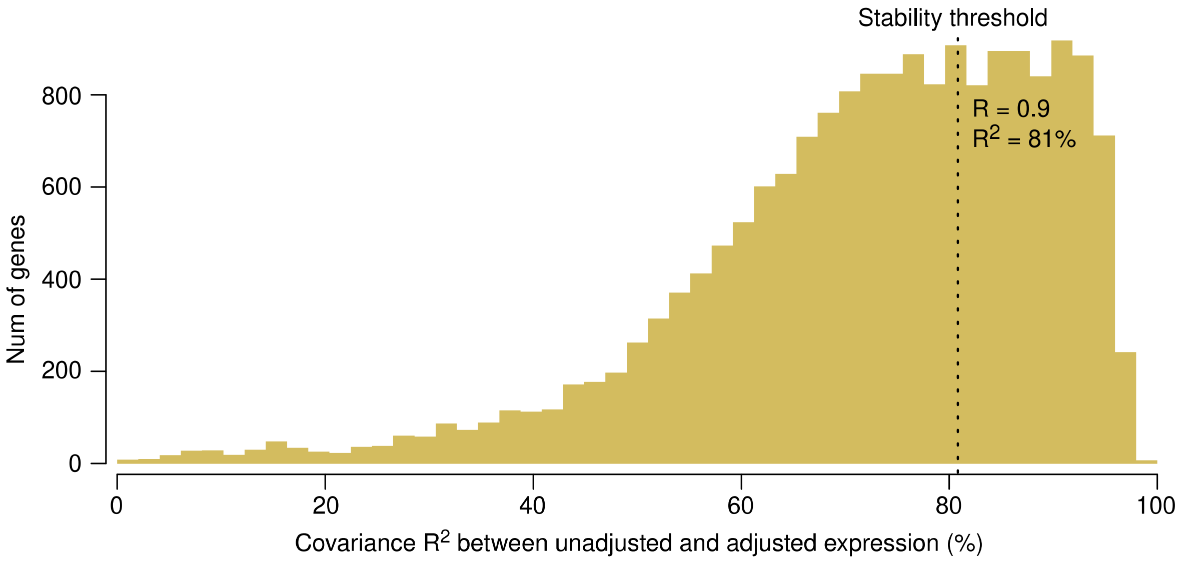

A summary of the impact of batch correction on gene expression levels. The histogram shows Pearson correlations for each gene between original log transformed read counts and read counts after batch correction. We then defined acceptable stability as $R > 0.9$, which led to the inclusion of 6,673 genes out of 18,503 for further statistical analyses.
